# Ten-Color flow cytometry reveals distinct patterns of expression of CD124 and CD126 by developing thymocytes

**DOI:** 10.1186/1471-2172-12-36

**Published:** 2011-06-20

**Authors:** Chibing Tan, Ashlee A Taylor, Matthew Z Coburn, Julie H Marino, C Justin Van De Wiele, T Kent Teague

**Affiliations:** 1 Department of Surgery, University of Oklahoma College of Medicine, Tulsa, OK, USA; 2 Department of Pharmaceutical Sciences, University of Oklahoma College of Pharmacy, Tulsa, OK, USA; 3 Department of Biochemistry and Microbiology, OSU Center for Health Sciences, Tulsa, OK, USA

## Abstract

**Background:**

We have developed a 12-parameter/10-color flow cytometric staining method for the simultaneous detection and characterization of 21 mouse thymocyte subpopulations that represent discreet stages of T cell development. To demonstrate the utility of this method, we assessed cytokine receptor expression on mouse thymocyte subsets. These experiments revealed distinct patterns of surface expression of receptors for the cytokines IL-4 and IL-6.

**Results:**

The IL-4 receptor α chain (CD124) was highly expressed on the earliest thymocyte subsets, then downregulated prior to T cell receptor β-selection and finally upregulated in the CD4/CD8 double positive cells prior to positive selection. The IL-6 receptor α chain (CD126) showed a different pattern of expression. It was expressed on the most mature subsets within the CD4 and CD8 single positive (SP) compartments and was absent on all other thymocytes with the exception of a very small cKit^-^CD4^-^CD8^- ^population. Intracellular staining of SP thymocytes for phosphorylated STAT-1 demonstrated that IL-6 signaling was confined to the most mature SP subsets.

**Conclusions:**

This 12-parameter staining methodology uses only commercially available fluorochrome-coupled monoclonal antibodies and therefore could be employed by any investigator with access to a 4-laser flow cytometer. This novel staining scheme allowed us to easily phenotype thymocyte subpopulations that span across development, from the early thymic progenitors (ETPs) to the most mature subsets of the CD4 and CD8 single positive populations.

## Background

**Figure 1:**
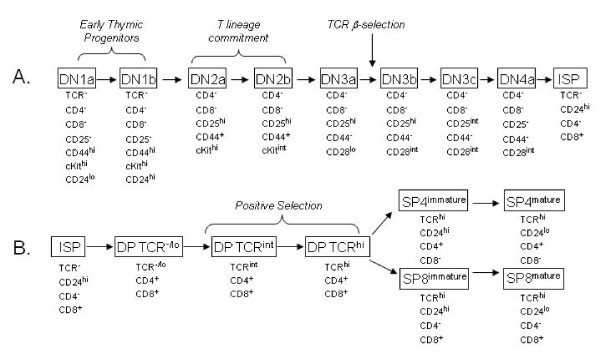
**Mouse thymocyte development scheme**. Mouse T cells develop in the thymus from early thymic progenitors (ETPs) to mature thymocytes that are single positive for CD4 (SP4) or CD8 (SP8). (A) shows the maturational pathway from the ETPs to the intermediate CD8 single positives (ISPs). (B) shows the maturation from the ISP stage to the most mature single positive stages. DP = double positive for CD4 and CD8, DN = double negative for CD4 and CD8, TCR = T cell receptor. Surface antigens used to discriminate the subsets and important developmental events are noted. Thymocyte subsets not directly in the T cell lineage are not depicted. Non-T-lineage markers (Lin) used for discrimination are also not depicted.

Recent studies, including fate mapping using OP9-DL1 stromal cells (reviewed in [1]), have allowed for finer discrimination and sequential ordering of a number of discreet thymocyte subpopulations. Due to the large number of different surface antigens used as markers for identification of each subset (Figure 1), it has become increasingly difficult to perform comprehensive phenotyping of mouse thymopoiesis by flow cytometry. Many investigators have been forced to rely on cell sorting prior to phenotype staining or to focus on either early or late development in order to assess subsets with finer resolution. We have focused on the development of phenotyping stains that target thymocytes across all of the major stages of maturation without the need for pre-sorting. As described below, these stages of maturation can be finely assessed using flow cytometric analysis of a number of surface antigens that are unique to each stage.

Traditionally, the major murine thymocyte compartments are defined by expression of the CD4 and CD8 antigens [2]. The most immature cells are negative for both antigens (DN), whereas cells of intermediate maturity are double positive (DP) for CD4 and CD8. The DP cells give rise to the most mature cells which are single positive for either CD8 (SP8) or CD4 (SP4) [3,4]. These four major compartments have been further divided into at least 18 discreet subpopulations as previously published by us and others [2,5-10], some of which are depicted in Figure 1. Important checkpoints that determine whether thymocytes live or die are noted in this figure as well. The first major checkpoint occurs in the DN3 compartment where only cells that successfully express a TCRβ chain are selected to continue maturation [11]. Cells in the DN3 compartment that are pre-or post-β-selection can be discriminated into DN3a and DN3b subsets, respectively, based on CD28 [10] or CD27 expression [12]. As depicted in Figure 1 and recently described by us using OP9-DL1 fate mapping assays [13], DN cells that are pre and post β-selection and those between β-selection and the DP stage can be finely resolved using CD25 and CD28 staining. It is in the DP compartment that positive selection occurs (reviewed in [14]) and this compartment can be divided into those cells that are negative/low for TCR expression, those that have intermediate TCR expression and those cells that are TCR high/CD69 high which are the cells that have just undergone positive selection. Finally, the small percentage of cells that mature into the SP4 and SP8 populations progressively lose expression of CD24 (heat stable antigen) until ready for entry into the periphery [15] and thus can be divided into semi-mature SP and mature SP subsets based on level of this antigen. It is during the SP stages that negative selection occurs (reviewed in [6]).

Throughout thymocyte development, the regulation of access and responsiveness to various cytokines is critical to maintaining normal thymopoiesis. For example, genetic defects in the common cytokine receptor γ chain results in X-linked severe combined immunodeficiency (SCID) [16]. IL-7 is known to be particularly important in modulating thymocyte survival through various checkpoints [17]. Furthermore, IL-7 deficient mice have reduced numbers of thymocytes as well as skewed ratios of α βversus γ δ T cells [18]. While less is known about the contribution of other cytokines to thymocyte development, engineered loss of the IL-6 receptor α chain (IL-6Rα) has a noticeable impact on thymopoiesis [19].

Here we describe the use of a panel of commercially available mAbs that can be employed to simultaneously identify and phenotype 21 thymocyte subsets that span across T cell development. These subsets include 18 subsets previously defined by others, and three subsets recently described by us[13]. We demonstrate the utility of this staining scheme by quantifying the expression of the IL-4Rα chain (CD124) and the IL-6Rα chain (CD126) in the thymocyte subpopulations.

## Results

### Ten color flow cytometry discriminates thymocyte subpopulations

**Figure 2:**
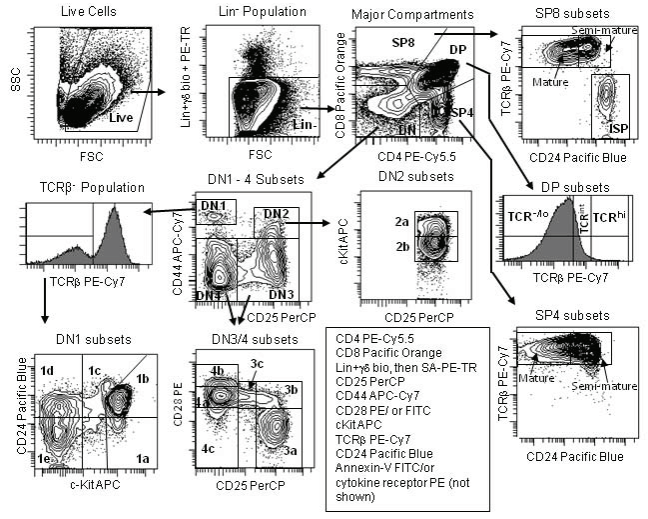
**Gating strategy used for the 10-color flow cytometric stain**. Thymocytes were phenotyped by flow cytometry with Annexin-V-FITC and mAbs against mouse CD4, CD8, CD25, CD28, CD44, CD117, CD24, TCR-β and either IL-4Rα (CD124) or IL-6Rα (CD126). Only singlets and Annexin-V negative cells were included in the analysis. Cells of other lineages were excluded using mAbs against the markers NK1.1, Ly6, Ter119, CD19, CD11b, and TCR-γδ. Arrows show the logical gating progression used to achieve discrimination of the 21 subsets. Forty million thymocytes were stained to generate this figure.

Figure 2 shows the 10-color staining and gating scheme we have developed to comprehensively discriminate thymocyte subpopulations across development using a single stain. This scheme leaves the PE channel open for assessment of the relative expression of an additional antigen in each of the subpopulations. The ratios of the major populations of DN, DP, SP4, and SP8 cells and relative staining patterns were similar to those previously published [20-22]. These populations were further parsed into the indicated subsets, which again showed expected staining patterns previously published with less comprehensive staining methods. Importantly, this staining scheme allowed for the discrimination of subsets from the earliest DN1 populations throughout development to the most mature SP populations, without the need for cell sorting or multiple stains (Figure 1). Table 1 shows the average percentages of each of the 21 subpopulations from 10 individual mice. Of note, the use of CD25 expression relative to CD28 expression not only discriminated between pre-β-selected and post-β-selected cells [10], but also showed a DN CD44 negative, CD28 positive, CD25 intermediate population that we recently defined as DN3c [13]. This staining scheme also illuminates DN4 subsets which we define as DN4a (CD25^-^CD28^int^), DN4b (CD25^-^CD28^hi^), and DN4c (CD25^-^CD28^-^) [13]. The DN4b cells express high levels of TCR and thus appear to be a small subset of mature thymocytes that fail to express CD4 and CD8 (data not shown). Our previous OP9-DL1 fate mapping assays showed that DN3c cells give rise to DN4a cells which represent preDP cells in the DN compartment[13]. The advantage of this polychromatic scheme is the ability to enumerate and characterize all these populations simultaneously without the need for pre-sorting or separate stains. We also validated the ten-color stain by comparing it with a simpler six-color stain that used different combinations of antibodies. As shown in Additional File 1 Figure S1, the distributions of the major thymocyte subsets were very similar between the two staining schemes.

**Table 1:** Relative distribution of the 21 thymocyte subsets*

	Average percentage (%)	STDEV (%)	Range (%)	Lower/upper 95% CI of mean (%)
**DN1a**	0.00107	0.000274	0.000881-0.00151	0.00073-0.00141

**DN1b**	0.0117	0.00214	0.00865-0.0146	0.00903-0.0143

**DN1c**	0.00115	0.000308	0.000713-0.00149	0.000772-0.00154

**DN1d**	0.00528	0.00089	0.00429-0.00650	0.00417-0.00638

**DN1e**	0.00510	0.00104	0.00391-0.00660	0.00381-0.00639

**DN2a**	0.0175	0.00357	0.0123-0.0205	0.0130-0.0219

**DN2b**	0.0769	0.00401	0.0725-0.0834	0.0720-0.0819

**DN3a**	1.205	0.145	1.053-1.420	1.0249-1.386

**DN3b**	0.179	0.0154	0.153-0.193	0.160-0.198

**DN3c**	0.170	0.0204	0.135-0.184	0.144-0.195

**DN4a**	1.528	0.296	1.197-1.789	1.160-1.894

**DN4b**	0.385	0.0879	0.290-0.494	0.276-0.495

**DN4c**	0.169	0.107	0.0675-0.290	0.0360-0.302

**ISP**	0.672	0.0892	0.552-0.731	0.561-0.783

**TCR^lo ^DP**	73.933	1.938	71.609-76.656	71.527-76.340

**TCR^int ^DP**	14.146	1.435	13.082-16.261	12.364-15.928

**TCR^hi ^DP**	1.113	0.141	0.944-1.265	0.938-1.288

**CD24^hi ^SP4**	3.862	0.216	3.504-4.0475	3.593-4.131

**CD24^lo ^SP4**	1.0966	0.0944	0.973-1.204	0.979-1.214

**CD24^hi ^SP8**	0.872	0.110	0.696-0.949	0.736-1.009

**CD24^lo ^SP8**	0.604	0.0235	0.582-0.638	0.575-0.633

* Mean percentages and SDs are derived from stains performed on thymuses from 10 individual C57BL/6 mice using ten million thymocytes from each mouse.

### Assessment of CD124 and CD126 expression during T cell development

**Figure 3:**
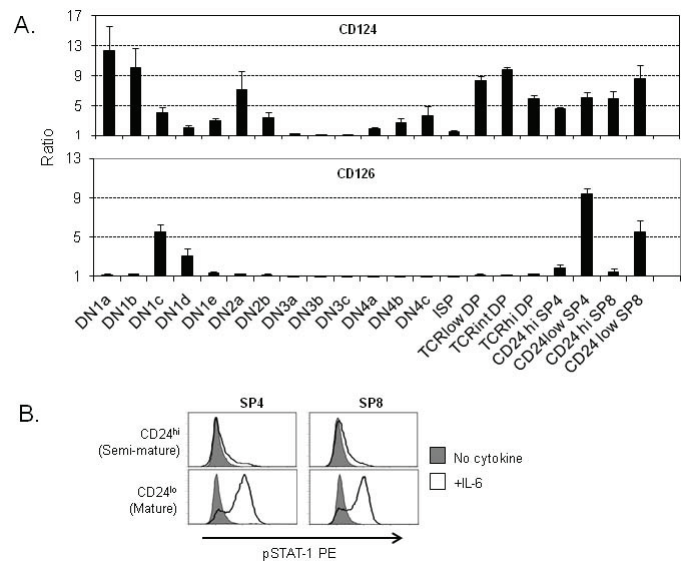
**Surface expression of CD124 and CD126, and IL-6 signaling**. The 10-color flow cytometric staining scheme shown in Figure 2 was used to evaluate expression of the cytokine receptors CD124 and CD126 (A). The FSC-adjusted MFI values for each receptor-specific stain were divided by the MFI values obtained from each corresponding isotype control stain to generate the ratios shown in the graph. A ratio of 1 indicates no change compared to isotype control. Bars represent the average MFI values from ten separate experiments and the error bars represent SD. Ten million cells were stained for each experiment shown in (A). In (B), surface staining of CD4, CD8 and CD24 was used to sort CD24^lo^SP4, CD24^hi^SP4, CD24^lo^SP8, and CD24^hi^SP8 populations. Sorted cells were subsequently treated with (open histograms) and without (shaded histograms) 25 ng/ml IL-6 as described in the methods section. Inducible IL-6 signaling was assessed by intracellular pSTAT-1-PE staining. The assay in (B) is representative of three experiments. 250,000 sort-purified cells from each indicated subset were used for the experiment shown.

To show the utility of the 10-color staining scheme, we assessed surface expression of two potentially important cytokine receptors across thymocyte development (Figure 3). The ratios of the median fluorescent intensities for CD124 (IL-4Rα) and CD126 (IL-6Rα) relative to the corresponding isotype controls, and adjusted for FSC differences, are shown in Figure 3A (and summarized in Additional File 2 Table S1). These stains revealed two distinct patterns of expression. CD124 expression was very high on DN1a and DN1b early T cell precursors (ETPs), whereas CD126 showed very low expression on the ETPs. As the cells progressed from ETPs to the DN2 populations, they retained expression of CD124 but expression of this receptor waned as the thymocytes approached the β-selection checkpoint (DN3a stage). As we have previously published, TCR^- ^and TCR^lo ^DP cells showed little expression of CD126 but high expression of CD124 [23]. The most mature populations of SP4 and SP8 cells expressed both cytokine receptors. Of particular note, CD126 was highly expressed only on the most mature SP4 and SP8 populations (CD24^lo ^cells) and two very small DN1 subsets (DN1d, DN1e) that are reportedly not efficient precursors to mature T cells [8].

### IL-6 signaling via pSTAT1 correlates with expression of IL-6Rα in the CD24^lo^SP4 and CD24^lo^SP8 populations

An IL-6 response assay was performed to determine whether the limited IL-6Rα (CD126) expression pattern shown in Figure 3A correlated with a similar pattern of responsiveness to IL-6 (Figure 3B). The CD24^hi^SP4, CD24^lo^SP4, CD24^hi^SP8, and CD24^lo^SP8 subsets were sort purified as described in the Materials and Methods. Cells were assessed for response to IL-6 as previously described [17,23]. Cells were treated for 20 minutes in the presence or absence of 25 ng/ml of IL-6 and responsiveness assessed by intracellular staining, using mAbs specific to the phosphorylated form of STAT1 (pSTAT1). As predicted from the pattern of surface expression of the IL-6Rα, IL-6 responsiveness was most evident in the mature CD24^lo^SP4 and CD24^lo^SP8 populations.

## Discussion

The 10-color stain described above allowed us to easily define the relative distribution of 21 discreet thymocyte subsets (Table 1) and to quantitate cytokine receptor surface expression across development at much finer resolution than previously reported. We have previously demonstrated that CD4-CD8-thymocytes are a mixed population with regard to IL-4-induced intracellular signaling as assessed by pSTAT5 [23]. In this study we further divided the DN compartment into 13 separate populations and a clearer picture emerged of IL-4Rα expression during early thymocyte development. The DN3a, DN3b, and the DN3c populations exhibited very little surface expression of IL-4Rα (Figure 3A), suggesting loss of IL-4 signaling occurs in T cell development before cells undergo β-selection. IL-4 receptor was upregulated in the more mature thymocyte populations (DP and SP cells). There is an apparent disconnect between the expression levels of the IL-4 receptor shown in Figure 3A and previous findings detailing the extent of thymocyte signaling via IL-4 [23,24]. We have previously shown that DP thymocytes have diminished levels of IL-4 responsiveness compared to SP cells [23], which may be due to suppression via SOCS-1 as reported by Yu et al. [24].

Within the standard αβ T cell development pathway, we show that IL-6Rα expression is restricted to only the most mature SP4 and SP8 populations (Figure 3A). These populations are defined as semi-mature and mature based on high or low expression of CD24 as previously described [7,15]. We also demonstrated that a small fraction of the DN1 compartment (DN1c, DN1d, and DN1e) also expresses this receptor, however, the subsets of DN1 that are reported to be efficient T cell progenitors (DN1a and DN1b) were very low or negative for IL-6Rα. Intracellular staining for IL-6-induced phosphorylation of STAT-1 (Figure 3B) and STAT-3 (data not shown) clearly indicated that only the most mature SP subsets were capable of responding to IL-6 stimulation. These data confirm and expand on the previous findings by Betz and Muller who first proposed IL-6Rα as a marker for mature thymocytes in mice [25]. They demonstrated that gp130 (IL-6 receptor signaling subunit) is expressed at consistent levels throughout thymocyte maturation while IL-6Rα is found only in the SP populations. They also showed that IL-6Rα^+ ^SP4 thymocytes represented a more mature population as they were not sensitive to hydrocortisone and were able to proliferate in response to TCR crosslinking, unlike the IL-6Rα^- ^SP4 population. Use of our novel staining method, which examined IL-6Rα at a higher resolution across development, confirmed IL-6Rα as a marker for mature SP thymocytes. Importantly, our experiments using sort purified cells showed that IL-6-induced STAT signalling is confined to the mature SP subsets as would be predicted by IL-6Rα staining.

The exact role of IL-6 in thymopoiesis is unknown; however, it may augment responsiveness of the mature populations to other cytokines. For example, Suda, et al. [26] demonstrated that IL-6 increased the proliferative response of SP4 and SP8 thymocytes to IL-2 and IL-4 *in vitro*. Additionally there is a significant loss of total thymocyte numbers in IL-6 deficient mice [19]. Sempowski et al. reported that in human thymus samples, IL-6 transcript increases progressively during aging [27]. Interestingly, they also reported that *in vivo *injection of mice with IL-6 over the course of 3 days led to thymic atrophy and particular loss of DP thymocytes, a population we find to express little, if any IL-6Rα. Given these observations it is clear that our understanding of the role of IL-6 within the thymus is incomplete and further study is warranted.

## Conclusions

The staining scheme that we present in this manuscript allowed us to simultaneously characterize 21 cell subsets that spanned T cell development in the thymus. We found distinct expression patterns of the receptors for IL-4 and IL-6 as thymocytes develop. Importantly, cell sorting was not necessary to pre-enrich the populations prior to analysis of surface receptor expression, which allowed for rapid phenotyping of thymopoiesis utilizing fewer mice. This method was designed to use only commercially available mAb-fluorochrome conjugates in order to allow any investigator with access to a four-laser flow cytometer the ability to employ the staining scheme to quickly and efficiently characterize mouse thymopoiesis in normal or genetically manipulated mice.

## Methods

### Flow Cytometer Setup and Data Acquisition

Flow cytometric data were acquired using a BD LSR II Flow Cytometer (BD Biosciences, San Jose, CA). This instrument is an air-cooled 4-laser benchtop flow cytometer with the ability to detect up to 12 individual fluorescent signals. The lasers and filters used were supplied with the instrument from BD Biosciences. The lasers used in this study were the following: solid state, 50 mW, 488 nm (blue); solid state, 50 mW, 405 nm (violet); solid state, 150 mW, 532 nm (green); helium-neon, 70 mW, 633 nm (red). The photomultiplier (PMT) setup is depicted in Additional File 3 Figure S2 and used a trigon configuration for blue, violet, and red laser paths and an octagon configuration for the green detector arrays. Dichroic long pass mirrors were used on the inner rings, and band pass filters used on outer rings. A 2-Blue, 2-Violet, 5-Green, 3-Red fluorochrome excitation configuration was used in this study (Additional File 3 Figure S2). Instrument performance was validated using BD Cytometer Setup and Tracking (CS&T) beads (part #: 910723, Lot ID: 75445, BD Biosciences, San Jose, CA). This validation was performed routinely and prior to each of the large phenotyping experiments. Triggering was set on the forward scatter (FSC) signal. FACS DIVA 6.0 software (BD Biosciences) was used to acquire digital data.

### Fluorochrome Compensations and PMT Settings

Although PMT settings and compensations should be adjusted for each instrument and stain setup, we include here the settings used for our instrument which were the following: the FSC setting included both area and width, with a threshold of 10,000, and a PMT Voltage of 315. The SSC setting included area with a PMT voltage of 300. All other PMT settings (log scale) were as follows: Pacific Blue = 380, APC = 545, APC-Cy7 = 568, FITC = 539, PerCP = 642, PE = 577, PE-Texas Red = 501, PE-Cy5.5 = 479, PE-Cy7 = 551, and Pacific Orange = 468. The PMT voltage settings were manually set using unstained cells as a reference. Each histogram was adjusted to allow a full view of the peak at approximately the same intensity for each fluorochrome.

Unstained and single fluorochrome-mAb stained thymocytes were used to establish baseline instrument application settings and compensation settings for each fluorochrome measured. To determine and validate compensation settings for fluorescence on small populations, cells of interest were sort enriched prior to use. Preliminary experiments were designed to first assess the staining patterns of small panels of mAbs and then the staining patterns observed in these smaller panels were compared with those previously observed in simple two-, three-, or four-color experiments. After comparable results were obtained, an additional interest marker was added, and separate "fluorescence minus one" (FMO) controls were prepared to ensure that the staining patterns and all subset populations of the original markers were not changed by the addition of the new marker. FMO control histograms are shown in Additional File 4 Figure S3. Using this sequence, the 10-color staining panel was eventually successfully validated.

### Cell Sorting

For the pSTAT assays shown in Figure 3B, the cell subsets indicated in the figure legend were sort-purified using a two-laser Moflo cell sorter (Beckman-Coulter, Fullerton, CA) and Summit v4.3 software. For the validation of compensation settings for the small DN thymocyte subsets, cells were stained with a single fluorochrome and sort-enriched using the MoFlo cell sorter prior to use on the LSR II. These cells were stained and positively enriched using one of the following mAb or mAb combinations: cKit-APC, CD25-PerCP, CD44-APC-Cy7, or Lin-biotin/TCR γδ-biotin followed by streptavidin-PE-Texas Red.

### Data Analysis

Data were analyzed using FACS DIVA 6.0 software (BD Biosciences). For all experiments, cell doublets and clusters were gated from the analysis using doublet discrimination (FSC width versus FSC area). Cell debris and small particles were excluded by gating out events with low forward scatter. The gating strategy is shown in Figure 2. CD4 and CD8 double positive and single positive populations that were negative for the non-T lineage markers (Lin) and TCR-γδ were further discriminated as follows: DP (CD4^+^CD8^+^), SP4 (TCR^+^CD4^+^CD8^-^), and SP8 (TCR^+^CD4^-^CD8^+^). DP TCR negative, DP TCR intermediate, and DP TCR high were separated by TCR-β chain (H57-597) expression. CD4^-^CD8^- ^(DN) populations were further defined as follows: DN1 (Lin^-^CD25^-^CD44^+^), DN2 (Lin^-^CD25^+^CD44^+^), DN3 (Lin^-^CD25^+^CD44^-^) and DN4 (Lin^-^CD25^-^CD44^-^). Five subsets of TCR negative DN1 thymocytes (DN1a-DN1e), as defined by c-Kit and CD24 expression were discriminated as previously demonstrated [8]. DN3 and DN4 were also further defined based on CD25 and CD28 expression. DN3 cells were divided into CD28 low/CD25 high (DN3a), CD28 intermediate/CD25 high (DN3b) and CD28 intermediate/CD25 intermediate (DN3c). DN4 cells were divided into CD28 intermediate (DN4a), CD28 high (DN4b), and CD28 low (DN4c) cells as described in the results section.

In Figure 3, median fluorescent intensity (MFI) ratios were calculated based on the FSC-adjusted MFI values of the indicated specific cytokine receptor stains divided by the FSC-adjusted MFI values from the corresponding isotype control stains. FSC adjustment (PE MFI/FSC MFI) was done in order to account for cell size differences in the different subsets. This also allowed for normalization across the multiple experiments. MFI ratio averages and standard deviations were calculated from a minimum of five separate experiments for each cytokine receptor. To do overlay plots, FCS data were exported, and analyzed by FlowJo (Tree Star, Inc, Ashland, OR). Statistical analysis was generated using GraphPad Prism v. 4.00 (La Jolla, CA).

### Mice

Mice used in this study were C57BL/6 females ranging from 6 to 8 weeks of age that were bred and housed at U.S.D.A. approved animal facilities located at the University of Oklahoma in Tulsa and the University of Tulsa. Animal care and all animal experiments were done in accordance with procedures outlined in the Guide for the Care and Use of Laboratory Animals (National Research Council). Protocols were reviewed and approved by the Institutional Animal Care and Use Committees of the University of Oklahoma Health Sciences Center and the University of Tulsa.

### Tissue harvest and cell staining

Thymuses were harvested and placed into complete tumor media (CTM) as previously described [28]. Thymuses were pressed through 70-μm nylon screens to generate single thymocyte suspensions [17]. Cells were treated with RBC lysis buffer (Sigma-Aldrich, St. Louis, MO), washed into CTM and counted using a hemocytometer. Cells were incubated with mAb against mouse CD16/CD32 (Fc Block, BD Biosciences) to block potential Fc-mediated binding and then stained at a density of 1 × 10^8 ^cell/ml with primary mAbs as indicated in Additional File 5 Table S2 for 45 minutes at 4°C in the dark. After two washes, the cells were further stained with Streptavidin PE-Texas Red (1:1600) for 30 minutes at 4°C in the dark. The monoclonal antibodies used in the 10-color stain experiments were individually tested. Optimal antibody titers (Additional File 5 Table S2) were determined for each monoclonal antibody and/or their secondary stain (Streptavidin PE-Texas Red) by staining thymocytes with mAb titers ranging from 1:50 to 1:1600, depending on the fluorochrome. For Annexin-V (Biolegend) staining, cells were washed twice after surface marker staining, then resuspended in Annexin-V binding buffer (Biolegend containing Annexin-V-FITC (1:100), and incubated for 15 minutes at room temperature in the dark.

### STAT phosphorylation assays

Assessment of IL-6-induced STAT-1 phosphorylation began with surface staining of total thymocytes with anti-CD4, CD8, and CD24 mAbs (Additional File 5 Table S2). These cells were sort-purified into the following populations: CD24^lo^SP4, CD24^hi^SP4, CD24^lo^SP8, and CD24^hi^SP8. Sorted cells were then incubated in the presence or absence of 25 ng/ml IL-6 (R&D Systems, Minneapolis, MN) for 20 minutes at 37°C. Intracellular stains were performed using Invitrogen/Caltag Fix & Perm reagents with an additional methanol step as previously described[23]. STAT-1 phosphorylation status was assessed with PE-conjugated anti-pSTAT1 (pY701) (BD Biosciences) using a PE-conjugated mouse IgG2a (BD Biosciences) as an isotype control.

## Authors' contributions

CT and TKT designed and coordinated the study and wrote/edited the manuscript. CT, AAT, and MZC carried out the immunoassays. CJV participated in the design of the study and edited the manuscript. JHM assisted in writing and editing the manuscript. All authors read and approved the final manuscript.

## Supplementary Material

Additional file 1 **Figure S1-Frequencies of the major thymocyte populations in the ten-color stain compared to a simpler six-color stain**. The six-color stain used was CD4-APC, CD8-pacific blue, CD25-PE, CD44-APC-Cy7, TCR-β-FITC, and T-lineage-biotin followed by SA-PE-TR. Frequencies of major thymocyte subsets are annotated in the figure. This figure represents one of five separate experiments that showed similar results. Click here for file

Additional file 2 **Table S1**. CD124 and CD126 expression across 21 thymocyte subsets from 10-color stain. Click here for file

Additional file 3 **Figure S2-The BD LSRII flow cytometer PMT/laser configuration**. The flow cytometer used in this study included: 2 PMTs/Blue laser; 2 PMTs/Violet laser; 5 PMTs/Green laser; 3 PMTs/Red laser configuration from the manufacturer. Light paths for each laser are depicted by arrows. Click here for file

Additional file 4 **Figure S3-Fluorescence minus one (FMO) controls**. The FMO histograms for each of the nine stains used to discriminate the thymocyte subsets. Click here for file

Additional file 5 **Table S2**. Staining reagents used in the 10-color stain. Click here for file
