# Supplementary figures and images for: Ten-Color flow cytometry reveals distinct patterns of expression of CD124 and CD126 by developing thymocytes
Source: BMC Immunol. 2011 Jun 20;12:36. doi: 10.1186/1471-2172-12-36 (PMC3130696; doi:10.1186/1471-2172-12-36)

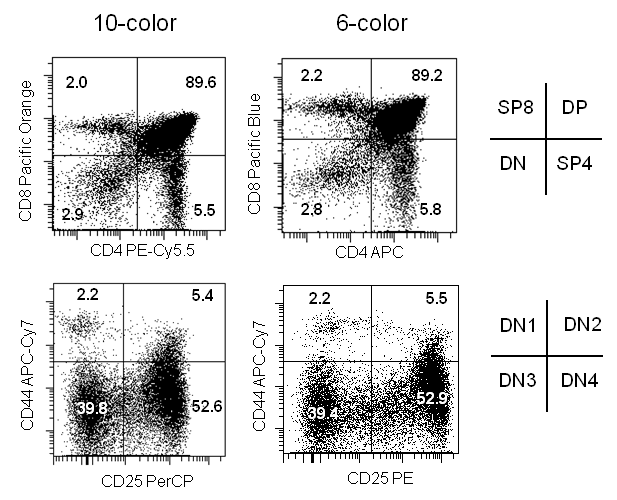

Supplement: Additional file 1 — Figure S1-Frequencies of the major thymocyte populations in the ten-color stain compared to a simpler six-color stain. The six-color stain used was CD4-APC, CD8-pacific blue, CD25-PE, CD44-APC-Cy7, TCR-β-FITC, and T-lineage-biotin followed by SA-PE-TR. Frequencies of major thymocyte subsets are annotated in the figure. This figure represents one of five separate experiments that showed similar results. [file 1471-2172-12-36-S1.TIFF]

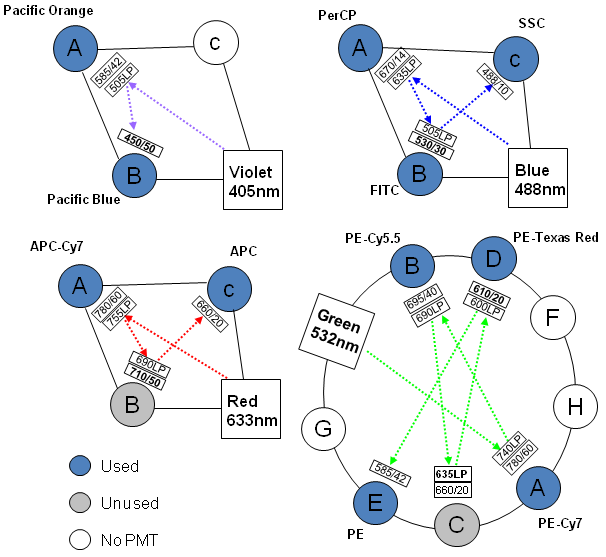

Supplement: Additional file 3 — Figure S2-The BD LSRII flow cytometer PMT/laser configuration. The flow cytometer used in this study included: 2 PMTs/Blue laser; 2 PMTs/Violet laser; 5 PMTs/Green laser; 3 PMTs/Red laser configuration from the manufacturer. Light paths for each laser are depicted by arrows. [file 1471-2172-12-36-S3.TIFF]

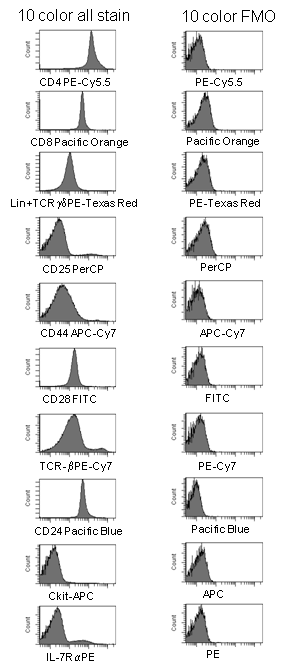

Supplement: Additional file 4 — Figure S3-Fluorescence minus one (FMO) controls. The FMO histograms for each of the nine stains used to discriminate the thymocyte subsets. [file 1471-2172-12-36-S4.TIFF]
