# Supplementary material for: Ten-Color flow cytometry reveals distinct patterns of expression of CD124 and CD126 by developing thymocytes
Source: BMC Immunol. 2011 Jun 20;12:36. doi: 10.1186/1471-2172-12-36 (PMC3130696; doi:10.1186/1471-2172-12-36)
Supplement: Additional file 2 — Table S1. CD124 and CD126 expression across 21 thymocyte subsets from 10-color stain. [file 1471-2172-12-36-S2.DOC]

**Table S1. CD124 and CD126 expression across 21 thymocyte subsets from 10-color stain**

|  | **CD124** | | **CD126** | |
| --- | --- | --- | --- | --- |
|  | **Median ratio** | **Frequency (%)** | **Median ratio** | **Frequency (%)** |
| **DN1a** | 12.41 ± 3.16 | 68.3 ± 5.6 | 1.04 ± 0.29 | 1.1 ± 0.5 |
| **DN1b** | 10.07 ± 2.61 | 79.1 ± 7.3 | 1.22 ± 0.06 | 2.3 ± 1.0 |
| **DN1c** | 4.10 ± 0.72 | 35.3 ± 2.5 | 5.50 ± 0.75 | 35.1 ± 6.9 |
| **DN1d** | 2.11 ± 0.37 | 19.1 ± 2.1 | 3.07 ± 0.80 | 34.2 ± 4.3 |
| **DN1e** | 2.98 ± 0.38 | 17.0 ± 2.3 | 1.36 ± 0.19 | 12.1 ± 5.1 |
| **DN2a** | 7.11 ± 2.52 | 71.1 ± 2.6 | 1.26 ± 0.03 | 0.9 ± 0.7 |
| **DN2b** | 3.37 ± 0.83 | 21.1 ± 2.1 | 1.13 ± 0.14 | 0.4 ± 0.3 |
| **DN3a** | 1.25 ± 0.07 | 0.3 ± 0.2 | 0.97 ± 0.06 | 0.2 ± 0.3 |
| **DN3b** | 1.21 ± 0.01 | 0.3 ± 0.3 | 0.96 ± 0.05 | 0.1 ± 0.1 |
| **DN3c** | 1.27 ± 0.05 | 1.2 ± 0.5 | 0.95 ± 0.06 | 0.1 ± 0.2 |
| **DN4a** | 1.93 ± 0.23 | 10.7 ± 2.3 | 0.95 ± 0.05 | 0.1 ± 0.1 |
| **DN4b** | 2.77 ± 0.54 | 39.2 ± 2.9 | 0.94 ± 0.04 | 0.2 ± 0.2 |
| **DN4c** | 3.75 ± 1.27 | 21.5 ± 3.1 | 0.95 ± 0.05 | 0.1 ± 0.1 |
| **ISP** | 1.60 ± 0.16 | 16.5 ± 1.2 | 0.98 ± 0.01 | 0.1 ± 0.1 |
| **TCR^low^ DP** | 8.38 ± 0.61 | 84.0 ± 4.8 | 1.11 ± 0.19 | 0.1 ± 0.1 |
| **TCR^int^ DP** | 9.82 ± 0.30 | 81.2 ± 3.9 | 1.11 ± 0.15 | 0.2 ± 0.1 |
| **TCR^high^ DP** | 6.03 ± 0.43 | 74.1 ± 3.5 | 1.20 ± 0.09 | 0.2 ± 0.2 |
| **CD24^high^ SP4** | 4.62 ± 0.22 | 48.9 ± 2.9 | 1.88 ± 0.45 | 16.8 ± 2.9 |
| **CD24^low^ SP4** | 6.05 ± 0.83 | 64.3 ± 5.6 | 9.26 ± 0.56 | 74.0 ± 8.5 |
| **CD24^high^ SP8** | 6.03 ± 0.91 | 58.2 ± 5.9 | 1.45 ± 0.35 | 2.9 ± 1.1 |
| **CD24^low^ SP8** | 8.62 ± 1.88 | 81.6 ± 3.7 | 5.31 ± 1.29 | 34.0 ± 5.9 |

Note: The median value is the ratio of receptor (median after FSC adjustment) vs the corresponding isotype control from 10 (CD124) and 11(CD126) independent experiments. Frequency values represent the percent staining positive for surface expression of indicated CD124 or CD126 relative to isotype control staining.
