# Supplementary material for: Ten-Color flow cytometry reveals distinct patterns of expression of CD124 and CD126 by developing thymocytes
Source: BMC Immunol. 2011 Jun 20;12:36. doi: 10.1186/1471-2172-12-36 (PMC3130696; doi:10.1186/1471-2172-12-36)
Supplement: Additional file 5 — Table S2. Staining reagents used in the 10-color stain. [file 1471-2172-12-36-S5.DOC]

**Table S2.** Staining reagents Used in the 10-Color Stain

| ANTIGEN | FLUOROCHROME | CLONE | SUPPLIER | STAINING TITER^+^ |
| --- | --- | --- | --- | --- |
| CD4 | PE-Cy5.5 | RM4-5 | Biolegend^c^ | 1:1000 |
| CD8 | Pacific Orange | 3B5 | Invitrogen/Caltag | 1:100 |
| CD25 | PerCP | PC61 | Biolegend | 1:200 |
| CD44 | APC-Cy7 | IM7 | Biolegend | 1:200 |
| Ckit (CD117) | APC | 2B8 | BD | 1:100 |
| CD28 | FITC or PE^d^ | E18 | Biolegend | 1:100 |
| CD24 | Pacific Blue | M1/69 | BD | 1:1000 |
| TCR-β Chain | PE-Cy7 | H57-597 | Biolegend | 1:400 |
| TCR-γδ | Biotin* | GL-3 | BD | 1:400 |
| Lineage cocktail | Biotin* | a | BD | 1:100 |
| IL-6Rα (CD126) | PE^b^ | D7715A7 | BD | 1:50 |
| IL-4Rα (CD124) | PE^b^ | mlL4R-M1 | BD | 1:50 |
| Rat IgG2b, K (Isotype control) | PE^b^ | A95-1 | BD | 1:50 |
| Rat IgG2a, K (Isotype control) | PE^b^ | R35-95 | BD | 1:50 |
| Annexin-V^d^ | FITC |  | Biolegend | 1:100 |

* Secondary stain: Streptavidin PE-Texas Red (1:1600)

^+^Titrations will change and should always be performed for each set of antibodies

and each individual instrument setup.

^a^ Lineage cocktail including: CD19 (1D3), CD11b (M1/70), NK1.1(PK136), TCR119(IY-76), and Gr-1(RB6-8C5)

^b^ PE channel is open for assessment of cytokine receptors or other surface or intracellular antigens

^c^Biolegend has discontinued this mAb-fluorochrome. It can now be purchased from Invitrogen/Caltag

^d^CD28 PE instead of FITC is used for Annexin-V stain
